# Trends and seasonal patterns in suicide and traffic accident mortality among youth in Hungary, 2000–2024: a nationwide population-based study

**DOI:** 10.1186/s12887-026-07419-9

**Published:** 2026-07-29

**Authors:** Zsanett Gabriella Urfi, Tibor András Nyári, Tamás Lantos

**Affiliations:** 1 Department of Medical Physics and Informatics, Albert Szent-Györgyi Medical School, University of Szeged, 9 Korányi Alley, Szeged, 6720 Hungary; 2 Doctoral School of Experimental and Preventive Medicine (PhD), Albert Szent-Györgyi Medical School, University of Szeged, 4 Béla Somogyi Street, Szeged, Hungary

**Keywords:** External-cause mortality, Suicide mortality, Traffic accident mortality, Adolescents, Young adults, Temporal trends, Seasonal variation, Regional disparities, Negative binomial regression, Hungary

## Abstract

**Background:**

External causes of death represent the leading causes of mortality among adolescents and young adults. This study aimed to investigate long-term, seasonal patterns, and regional disparities in mortality from external causes among individuals aged 15–24 years in Hungary between 2000 and 2024, including comparisons between the pre-pandemic (2000–2019) and pandemic (2020–2024) periods.

**Methods:**

Nationwide data on monthly and annual mortality from external causes (ICD-10: V01–Y89) were obtained from the Dissemination Database of the Hungarian Central Statistical Office for 2000–2024. Analyses focused on the two leading causes of external mortality in this age group: suicide and self-harm (X60–X84, Y87.0) and traffic accidents (V01–V99). Mortality rates were calculated using corresponding population data stratified by sex, age group, and region. Annual and seasonal trends were analysed using negative binomial regression models with population offsets. Results are presented as incidence rate ratios (IRRs) with 95% confidence intervals (95% CIs).

**Results:**

Between 2000 and 2024, 7,209 deaths from external causes were recorded among individuals aged 15–24 years (79% males). Significant declining trends were observed in overall external-cause mortality (IRR = 0.97; 95% CI: 0.96–0.97; *p* < 0.001), suicide (IRR = 0.98; 95% CI: 0.97–0.98; *p* < 0.001), and traffic accident mortality (IRR = 0.95; 95% CI: 0.94–0.95; *p* < 0.001). Seasonal variation was significant for overall external-cause mortality, with a peak in July (*p* < 0.001), and for suicide and traffic accident mortality, peaking in May (*p* < 0.001) and August (*p* < 0.001), respectively. Regional disparities were pronounced, with the highest mortality rates consistently observed in the Southern Great Plain region. Mortality patterns during the COVID-19 period did not substantially deviate from pre-pandemic trends.

**Conclusions:**

Although mortality from external causes among Hungarian youth declined significantly over the past 25 years, substantial gender and regional inequalities persist. The marked summer peak in traffic accident mortality and the elevated risk in specific regions highlight the need for targeted, seasonally sensitive prevention strategies.

**Supplementary Information:**

The online version contains supplementary material available at https://doi.org/10.1186/s12887-026-07419-9.

## Background

Hungary has long ranked among the countries with the highest mortality rates within the European Union [1]. Although cardiovascular diseases and cancers account for the majority of deaths in the total population, the pattern of mortality among adolescents and young adults differs substantially [2]. According to mortality data from the Hungarian Central Statistical Office (HCSO), external causes of death account for approximately 60% of deaths among individuals aged 15–24 years and traffic accidents and suicide together constitute nearly three-quarters of external-cause deaths in this age group [2].

External causes of death constitute a heterogeneous group of events, including unintentional injuries, intentional self-harm (suicide), assault, and injuries of undetermined intent, as classified by the International Classification of Diseases, 10th Revision (ICD-10) [3]. Among young people, these deaths are particularly tragic because they occur at the beginning of the life course and are largely preventable. Beyond the immediate loss of life, they generate profound social, psychological, and economic consequences for families and communities. Understanding their temporal and spatial distribution is therefore of major public health importance.

Suicide remains a significant public health concern worldwide and has consistently ranked among the leading causes of death in young people [4, 5]. Hungary historically exhibited one of the highest suicide rates in Europe, although a long-term decline has been observed over recent decades [6, 7]. Traffic accidents similarly represent a major source of premature mortality among youth [8], particularly among young males, who are more likely to engage in high-risk behaviours [9]. In the Hungarian population aged 15–24 years, suicide and traffic accidents constitute the two leading causes of external-cause mortality and together account for the majority of deaths from external causes [2]. Examining these causes in parallel may therefore provide a more comprehensive understanding of preventable mortality patterns among Hungarian youth.

Previous Hungarian studies have primarily focused on overall suicide trends or adult populations. Mortality patterns among children under 15 years have recently been analysed in detail, highlighting the predominance of traffic-related deaths in that younger age group [10]. To our knowledge, no study has yet extended a similarly comprehensive analysis to the 15–24-year age group, despite the distinct developmental, behavioural, and social characteristics of this life stage. Consequently, comprehensive investigations of long-term trends, seasonal variation, and regional disparities specifically in the 15–24-year age group remain limited. Moreover, little is known about how these patterns evolved during the COVID-19 pandemic period in Hungary.

Given that the majority of deaths among individuals aged 15–24 years are attributable to external causes, a detailed examination of their annual trends, seasonal fluctuations, and territorial distribution is essential. The present nationwide population-based study, therefore, aimed to investigate mortality from all external causes, with particular emphasis on suicide and traffic accidents, among Hungarian youth between 2000 and 2024. In addition, we compared patterns observed during the pre-pandemic (2000–2019) and pandemic (2020–2024) periods to assess potential deviations associated with COVID-19-related societal changes.

## Materials and methods

### Study design and data sources

We conducted a nationwide, population-based ecological study examining mortality from external causes among individuals aged 15–24 years in Hungary between 1 January 2000 and 31 December 2024. Mortality data were obtained from the publicly accessible Dissemination Database of the HCSO (2024) [2]. Causes of death were classified according to the International Classification of Diseases, 10th revision (ICD-10) [3]. External causes were defined using codes V01–Y89. Within this category, the two most frequent causes in this age group – traffic accidents (V01–V99) and suicides and self-harm (X60–X84, Y87.0) – were analysed in detail.

Monthly and annual numbers of deaths were retrieved stratified by sex, five-year age group (15–19 and 20–24 years), and European Union’s Nomenclature of Territorial Units for Statistics, level 2 (NUTS2) region. Corresponding annual population data were obtained from the same database. As official monthly population data for the 15–24-year age group were not available, monthly population denominators were estimated by interpolating between annual mid-year population figures and adjusting for monthly counts of births and deaths. This approach allowed approximation of the population at risk for seasonal analyses.

### Outcome measures and standardisation

Age-specific mortality rates were calculated separately for the 15–19 and 20–24-year age groups. To facilitate regional comparisons and account for potential differences in age structure across regions, age-standardised mortality rates were computed using the direct standardisation method with the European Standard Population (ESP 2013) as the reference [11].

### Territorial classification

Regional analyses were performed according to the European Union’s NUTS2 territorial classification system [12]. During the study period, Hungary comprised eight NUTS2 regions: Budapest (HU11), Pest County (HU12), Central Transdanubia (HU21), Western Transdanubia (HU22), Southern Transdanubia (HU23), Northern Hungary (HU31), Northern Great Plain (HU32), and Southern Great Plain (HU33). Regional mortality rates were directly standardised using the ESP to ensure comparability.

### Statistical analysis

Annual mortality trends were analysed using negative binomial regression models. This modelling approach was chosen because overdispersion may occur in count data when the variance exceeds the mean, thereby violating the assumptions of standard Poisson regression. In such situations, the negative binomial model, which can be interpreted as a Poisson–Gamma mixture, provides a more appropriate fit [13]. The logarithm of the population size was included as an offset term in all models to estimate mortality rate ratios. Results are presented as incidence rate ratios (IRRs) with 95% confidence intervals (95% CIs). To further characterise temporal changes in sex-specific mortality rate ratios, Joinpoint regression analysis was performed [14]. Annual percent changes (APCs) and statistically significant joinpoints were estimated using the Joinpoint Regression Program (version 4.9.1.0; National Cancer Institute, Bethesda, MD, USA).

Seasonal variation was examined using monthly death counts within a negative binomial regression framework, including month as a categorical variable. To confirm cyclic seasonal patterns, geometric methods were applied, namely the Edwards test [15], and its generalisation, the Walter–Elwood method [16]. These complementary approaches allowed assessment of sinusoidal seasonality in mortality data.

All analyses were conducted for overall external-cause mortality (V01–Y89) and separately for suicide and self-harm (X60–X84, Y87.0) and traffic accident mortality (V01–V99). Subgroup analyses were performed by sex and age group. Statistical significance was defined as a two-sided *p*-value below 0.05. All statistical analyses were carried out using STATA version 16.1 (StataCorp, College Station, TX, USA).

### Ethical considerations

The study was based exclusively on anonymised, aggregated, publicly available data provided by the HCSO. No individual-level identifiers were used, and therefore, ethical approval and informed consent were not required under national regulations.

## Results

### Overall mortality patterns

Between 2000 and 2024, a total of 7,209 deaths from external causes occurred among individuals aged 15–24 years in Hungary. The majority of these deaths were recorded among males (*n* = 5,700; 79%), while females accounted for 1,509 deaths (21%) (Table 1). Suicides represented 2,382 deaths (33% of all external-cause deaths) (Table 2), and traffic accidents accounted for 2,993 deaths (41.5%) (Table 3). Together, these two causes were responsible for nearly three-quarters of all external-cause mortality in this age group.

**Table 1:** Deaths from external causes among youth, 2000–2024 (*n* = 7209 total deaths)

Subgroup	Youth deaths from external causes (*n* = 7209)		
	Number of deaths	Proportion (%)	Rate per 100,000
Gender			
Male	5700	79.1	37.27
Female	1509	20.9	10.40
Age Group			
15–19 years	2623	36.4	18.66
20–24 years	4586	63.6	29.22
Nuts2 Region			
Budapest	826	11.5	17.61
Pest County	859	11.9	24.04
Central Transdanubia	758	10.5	23.04
West. Transdanubia	595	8.3	20.57
South. Transdanubia	690	9.6	24.73
Northern Hungary	958	13.3	25.49
Northern Great Plain	1154	16.0	23.62
Southern Great Plain	1046	14.5	26.73
Unspecified*	323	4.5	

*Homeless, foreigner, or unknown

**Table 2:** Suicide deaths among youth, 2000–2024 (*n* = 2382 total number of suicides)

Subgroup	Youth suicide deaths (*n* = 2382)		
	Number of deaths	Proportion (%)	Rate per 100,000
Gender			
Male	1952	81.9	12.76
Female	430	18.1	2.96
Age Group			
15–19 years	826	34.7	5.88
20–24 years	1556	65.3	9.91
Nuts2 Region			
Budapest	308	12.9	6.55
Pest County	272	11.4	7.62
Central Transdanubia	237	9.9	7.19
West. Transdanubia	181	7.6	6.26
South. Transdanubia	223	9.4	7.99
Northern Hungary	349	14.7	9.29
Northern Great Plain	402	16.9	8.23
Southern Great Plain	366	15.4	9.35
Unspecified*	44	1.8	

*Homeless, foreigner, or unknown

**Table 3:** Deaths from traffic accidents among youth, 2000–2024 (*n* = 2993 total deaths from traffic accidents)

Subgroup	Youth deaths from traffic accidents (*n* = 2993)		
	Number of deaths	Proportion (%)	Rate per 100,000
Gender			
Male	2297	76.7	15.02
Female	696	23.3	4.79
Age Group			
15–19 years	1057	35.3	7.52
20–24 years	1936	64.7	12.34
Nuts2 Region			
Budapest	230	7.7	4.90
Pest County	388	13.0	10.86
Central Transdanubia	343	11.5	10.43
West. Transdanubia	294	9.8	10.16
South. Transdanubia	310	10.4	11.11
Northern Hungary	324	10.8	8.63
Northern Great Plain	466	15.6	9.54
Southern Great Plain	460	15.4	11.75
Unspecified*	178	5.9	

*Homeless, foreigner, or unknown

The male-to-female mortality rate ratio for all external causes was 3.59 (95% CI: 3.39–3.81). The corresponding mortality rate ratios were 4.32 (95% CI: 3.89–4.79) for suicide and 3.13 (95% CI: 2.88–3.41) for traffic accidents (*Additional file 1: Supplementary Tables S1–S3*). Age differences were also marked: individuals aged 20–24 years exhibited a significantly higher mortality risk than those aged 15–19 years (IRR = 1.57; 95% CI: 1.49–1.64; *p* < 0.001).

Throughout the study period, suicide mortality remained consistently higher among males than females. The annual male-to-female suicide mortality rate ratio averaged 4.47 and ranged from 2.52 to 8.51. Joinpoint regression demonstrated temporal variation in the magnitude of this disparity, with no significant change between 2000 and 2014, a significant decline between 2014 and 2019, and a significant increase thereafter (Additional file 2: Supplementary Figure S1).

The age-standardised mortality rate for all external causes over the study period was 24.17 per 100,000 population. The corresponding age-standardised rates were 7.98 per 100,000 for suicide and 10.06 per 100,000 for traffic accidents.

### Regional distribution

**Fig. 1:**
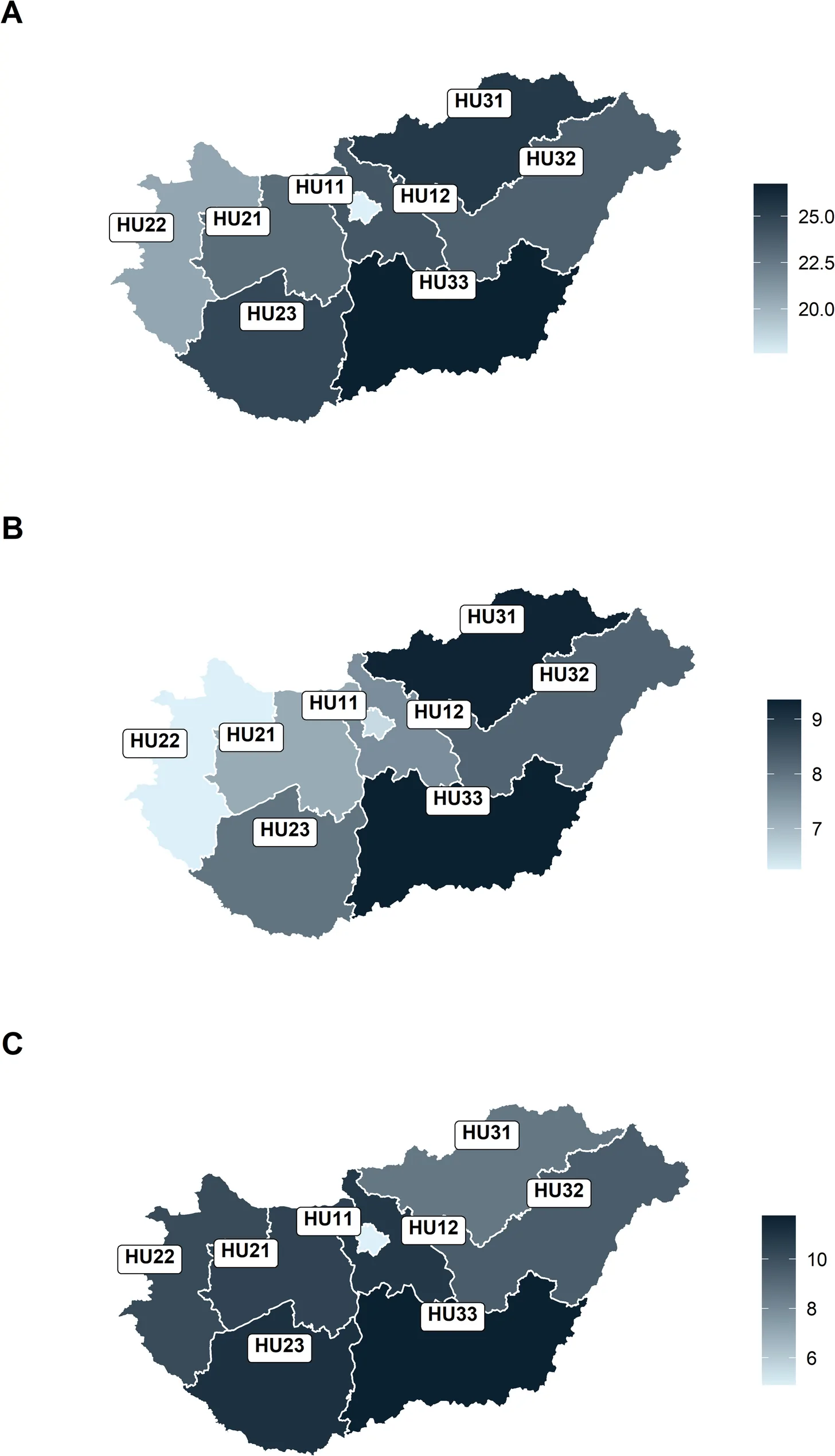
Geographical distribution of mortality from all external causes (**A**), suicide (**B**), and traffic accidents (**C**) among individuals aged 15–24 years in Hungary, 2000–2024. Maps show age-standardised mortality rates per 100,000 population based on the European Standard Population (ESP 2013)

Substantial regional variation was observed in mortality from external causes. Directly standardised mortality rates ranged from 17.61 to 26.73 per 100,000 individuals aged 15–24 years across NUTS2 regions, with the lowest rate observed in Budapest and the highest in the Southern Great Plain (Fig. 1A).

A similar territorial pattern characterised suicide mortality. Age-standardised suicide rates ranged from 6.26 to 9.35 per 100,000, with the highest values in the Southern Great Plain and Northern Hungary and the lowest in Western Transdanubia and Budapest (Fig. 1B).

Traffic accident mortality also displayed pronounced regional differences. Age-standardised rates ranged from 4.9 to 11.75 per 100,000, with Budapest showing the lowest rate and the Southern Great Plain the highest (Fig. 1C).

Sex-specific regional analyses showed that regional inequalities were more consistently evident among males than females (Additional file 2: Supplementary Figure S2; Additional file 1: Supplementary Tables S1–S3). Southern Great Plain was generally among the regions with the highest mortality rates in both sexes, particularly for all external-cause mortality and traffic accident mortality. In contrast, regional differences in suicide mortality were apparent primarily among males, whereas no region differed significantly from Budapest among females.

### Annual trends

**Fig. 2:**
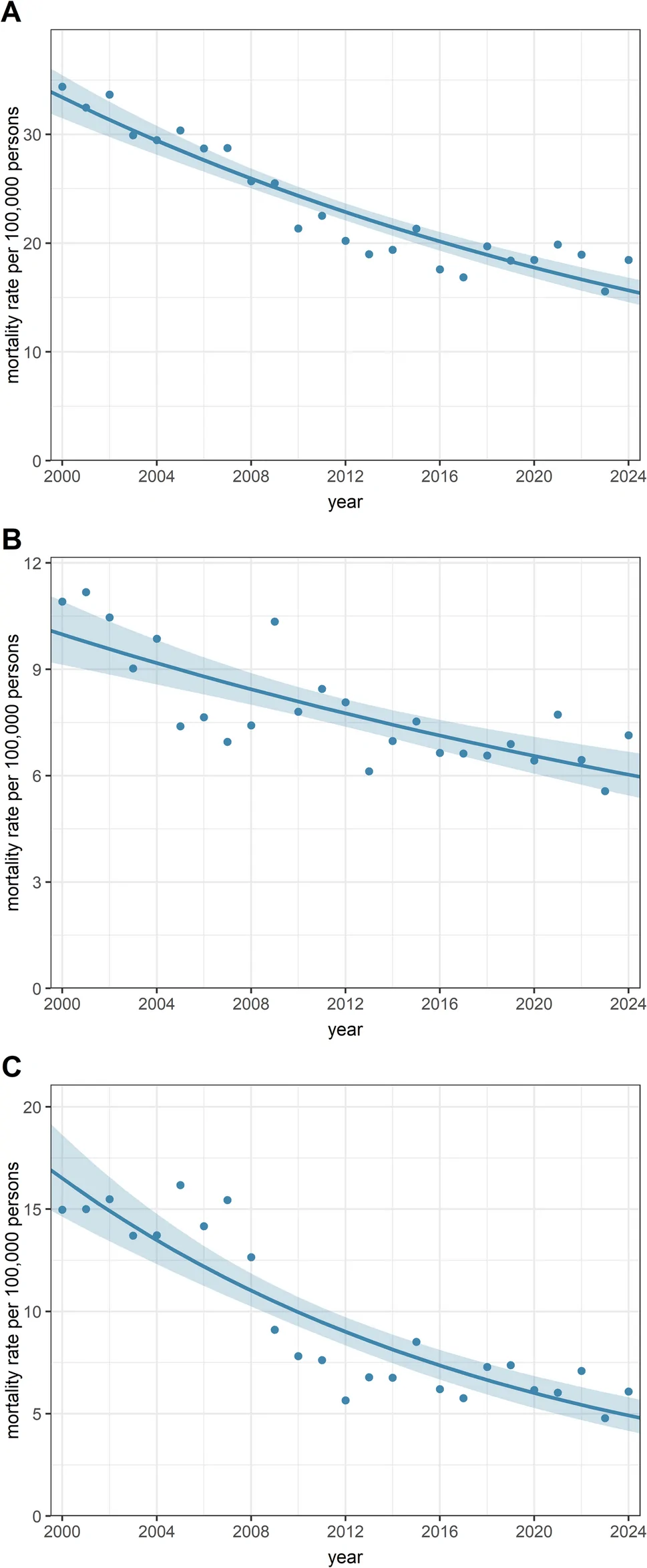
Annual trends of mortality from all external causes (**A)**, suicide (**B**), and traffic accidents (**C**) among individuals aged 15–24 years in Hungary, 2000–2024. Points represent annual mortality rates; solid lines represent fitted negative binomial regression trends with 95% confidence intervals

A significant decreasing trend was observed in mortality from all external causes over the 25-year study period (IRR = 0.97; 95% CI: 0.96–0.97; *p* < 0.001) (Fig. 2A). Suicide mortality also declined significantly (IRR = 0.98; 95% CI: 0.97–0.98; *p* < 0.001) (Fig. 2B), as did traffic accident mortality, which demonstrated the steepest reduction (IRR = 0.95; 95% CI: 0.94–0.95; *p* < 0.001) (Fig. 2C).

Sex-specific analyses demonstrated broadly similar temporal patterns among males and females (Additional file 2: Supplementary Figure S3). Mortality from all external causes, suicide, and traffic accidents declined significantly in both sexes, although absolute mortality rates remained substantially higher among males throughout the study period. The male-to-female suicide mortality rate ratio averaged 4.47 and exhibited significant temporal variation, with a decline between 2014 and 2019 followed by an increase thereafter.

Despite these long-term declines, mortality numbers for both suicide and traffic accidents showed relative stabilisation during the final five years of the study period (Figs. 2B–C). During the COVID-19 restriction period (2020–2021), external-cause mortality continued to decline in the 15–19-year age group. In contrast, mortality in the 20–24-year group remained largely stable during this period and returned to the pre-pandemic trajectory following the lifting of restrictions.

### Seasonal patterns

**Fig. 3:**
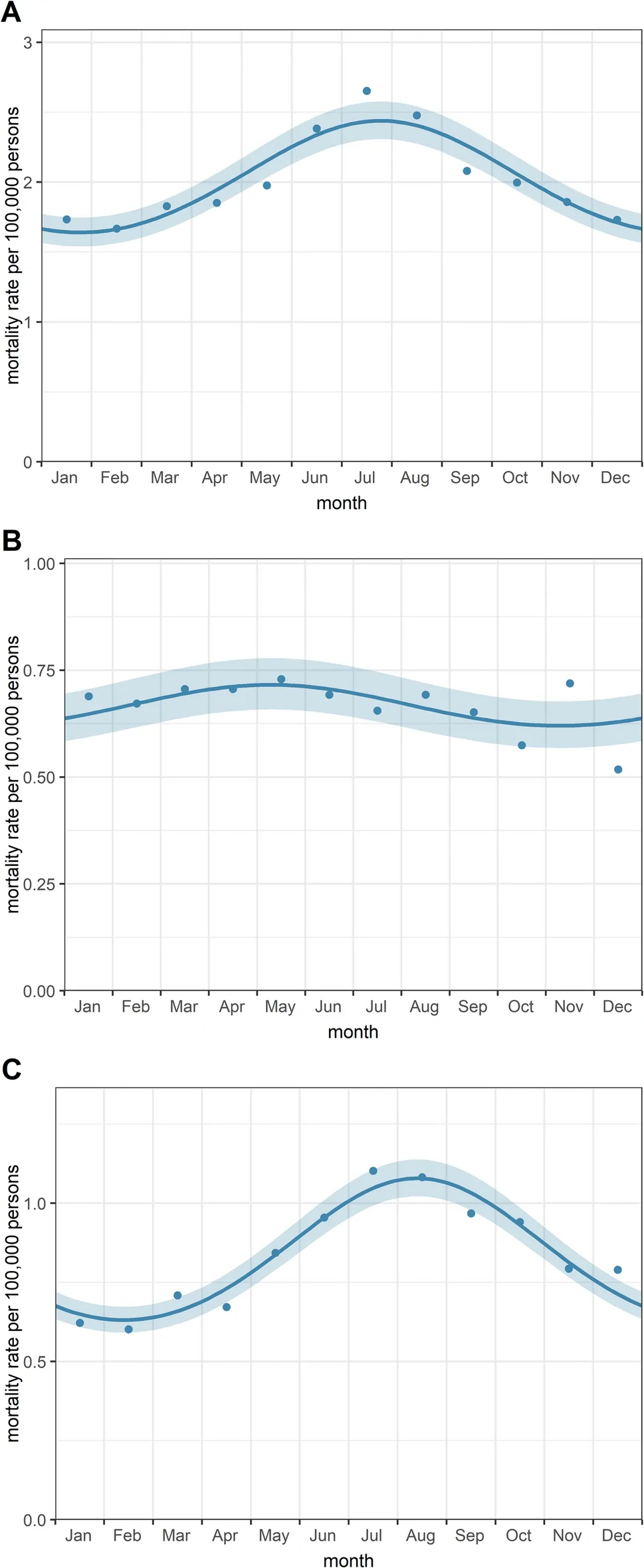
Seasonal patterns of mortality from all external causes (**A**), suicide (**B**), and traffic accidents (**C**) among individuals aged 15–24 years in Hungary, 2000–2024. Points represent monthly mortality rates; curves represent fitted negative binomial regression seasonal trends with 95% confidence intervals

Seasonal analysis revealed a significant cyclic pattern for overall external-cause mortality, with a peak occurring in July (*p* < 0.001) (Fig. 3A). Traffic accident mortality also demonstrated a pronounced pattern, with a peak in August across both sexes (*p* < 0.001) (Fig. 3C). In contrast, suicide mortality showed a significant (*p* = 0.038) seasonal pattern with a peak in May in this age group (Fig. 3B).

Sex-specific analyses revealed that significant seasonality was present among males for all external-cause mortality, suicide mortality, and traffic accident mortality (Additional file 2: Supplementary Figure S4). Among females, a significant seasonal pattern was observed only for traffic accident mortality, whereas seasonality was not statistically significant for either all external-cause mortality or suicide mortality.

Taken together, these findings indicate that although external-cause mortality among Hungarian youth has declined substantially over the past 25 years, pronounced gender differences, regional disparities, and seasonal concentration of traffic-related deaths persist.

## Discussion

### Main findings

This nationwide, population-based study provides a comprehensive 25-year overview of mortality from external causes among individuals aged 15–24 years in Hungary. Although a significant long-term decline was observed in overall external-cause mortality, suicide, and traffic accident deaths, external causes remain the dominant contributors to mortality in this age group. Marked gender inequalities, persistent regional disparities, and a pronounced seasonal peak in traffic accident mortality highlight important structural and behavioural determinants of risk.

### Comparison with other studies

Suicide remains a major global public health concern. According to the World Health Organization, suicide was the third leading cause of death among young people aged 15–29 years worldwide [5]. The multifactorial nature of suicidal behaviour – shaped by societal, psychological, and biological determinants – has long been emphasised [17]. Our findings are consistent with this international evidence, particularly regarding the marked male predominance in suicide mortality.

The approximately 4.5-fold higher suicide mortality observed among young Hungarian males reflects the well-documented gender paradox in suicidal behaviour: although suicide attempts are more frequent among females, suicide deaths are more common among males [17]. Similarly, the higher mortality risk observed in the 20–24-year age group compared with the 15–19-year group aligns with European findings reported by Bertuccio et al., who demonstrated substantially elevated suicide rates in emerging adulthood [4].

The significant downward trend in suicide mortality identified in our study is also consistent with broader European patterns [4]. Nevertheless, despite long-term improvements, Hungarian suicide mortality remains comparatively high within the European context.

Several factors may have contributed to the long-term decline in suicide mortality observed among Hungarian youth. Previous studies have suggested that improvements in the recognition and treatment of mood disorders, greater availability of psychiatric care, and increasing use of evidence-based pharmacological treatment may have played an important role in reducing suicide mortality in Hungary [6, 7]. Broader societal changes, including improving living standards and reductions in unemployment and smoking prevalence, have also been proposed as contributors to the favourable national trend [7]. Furthermore, international evidence indicates that comprehensive suicide prevention strategies, improved crisis intervention services, and enhanced access to mental health care can contribute to reductions in suicide mortality [5, 18]. However, the ecological design of the present study does not permit direct assessment of the relative contribution of these factors.

Seasonal variation in suicide has previously been reported in Hungary. Earlier national studies described a late spring–early summer peak, particularly in May–June, in the general population [19, 20]. In addition, analyses of long-term national data suggested that although seasonal variation in suicide remained evident in Hungary, its strength appeared to weaken over time [21].

In addition to these findings in broader age groups, our study has detected a significant seasonal pattern with a peak in May in suicide mortality among individuals aged 15–24 years. These findings are broadly consistent with previous Hungarian observations [19–21], although the seasonal effect appeared relatively modest in the present youth population. It is also possible that developmental and social characteristics unique to adolescence and emerging adulthood modify susceptibility to seasonal influences observed in older populations. Analyses of long-term national data have suggested that although seasonal variation in suicide remains observable in Hungary, its magnitude has weakened over recent decades [21]. The relatively modest magnitude of the seasonal effect observed in the present youth population may therefore reflect either age-specific differences or a continued attenuation of seasonal effects in more recent years. Interestingly, significant seasonal variation in suicide mortality was observed among males but not females. Although the relatively small number of female suicide deaths may have reduced statistical power, this finding is consistent with previous observations suggesting that seasonal fluctuations in suicide may be more pronounced among males than females [22].

By contrast, traffic accident mortality demonstrated a pronounced seasonal peak in August. This pattern is consistent with earlier Hungarian findings in children [10], and aligns with the broader seasonal concentration of accidental deaths reported in the total population [21]. Increased summer mobility, recreational travel, higher traffic density, and alcohol-related risk behaviours likely contribute to this concentration of mortality during holiday periods. In contrast to suicide mortality, significant seasonality in traffic accident mortality was evident in both sexes, suggesting that seasonal increases in exposure to traffic-related risks affect young males and females alike.

### Regional disparities and life-stage continuity

The pronounced territorial gradient identified in this study mirrors earlier Hungarian findings. Lantos et al. documented substantial regional inequalities in adult suicide mortality, with higher rates in the Great Plain regions and lower rates in Budapest [20]. Our results demonstrate that a comparable pattern characterises the youth population. The Southern Great Plain region exhibited consistently the highest mortality rates for both suicide and traffic accidents, whereas Budapest showed by far the lowest rate for traffic accidents and one of the lowest rates for suicide.

Comparable regional patterns were also observed among children under 15 years of age [10], particularly in traffic accident mortality. The persistence of this regional gradient across adjacent life stages suggests that enduring structural determinants – such as socioeconomic disadvantage, rurality, infrastructure characteristics, and healthcare accessibility – may contribute to the observed disparities.

### Trends over time and the COVID-19 period

The overall decline in mortality from external causes over the 25-year study period likely reflects the combined influence of improvements in road safety legislation, vehicle technology, and broader suicide prevention efforts. Previous analyses of the total Hungarian population demonstrated a similarly pronounced decrease in external-cause mortality between 1995 and 2014 [21], indicating that the favourable trajectory observed in our youth cohort forms part of a longer-term national pattern.

In particular, Lantos et al. identified a marked reduction in traffic accident mortality after 2007, temporally associated with substantial amendments to Hungarian traffic legislation introduced in 2008 [21]. These amendments included the implementation of a strict “zero tolerance” policy for drunk driving and intensified enforcement measures. The extensive media coverage accompanying these regulatory changes may have further strengthened their preventive impact. Furthermore, Hungary’s accession to the European Union in 2004 and the subsequent harmonisation of vehicle safety regulations – such as mandatory installation of seat belt systems in newly marketed vehicles – were also suggested as structural contributors to the decline in traffic accident mortality [21]. Additional contributors may include advances in vehicle safety technologies, improvements in road infrastructure, enhanced trauma and emergency medical care, and increased public awareness of road safety. Similar factors have been proposed to explain long-term reductions in traffic-related mortality across Europe [23, 24].

However, the relative stabilisation observed in recent years suggests that earlier gains may be plateauing, underscoring the need for renewed preventive efforts.

During the COVID-19 restriction period, no substantial deviation from pre-existing mortality trends was observed at the national level in the 15–24-year age group. Although mobility restrictions may have temporarily reduced exposure to traffic risks, psychosocial stressors associated with the pandemic did not translate into a detectable surge in suicide mortality in aggregate data. Nevertheless, national-level stability does not exclude increased vulnerability in specific subgroups.

### Joint consideration of suicide and traffic accidents

The parallel analysis of suicide and traffic accident mortality represents a particular strength of this study because these two causes accounted for nearly three-quarters of all external-cause deaths among Hungarian youth. Examining them together provides a comprehensive overview of the principal preventable causes of premature mortality in this age group and may help inform integrated prevention strategies.

### Public health implications

Suicide prevention requires a multifaceted strategy addressing distal, intermediate, and proximal risk factors. Hungarian literature has emphasised the importance of protective factors such as stable family environments, social support, and access to mental health care [6, 25]. Youth-focused crisis services, including the Hungarian Blue Line, also play a critical preventive role.

The present findings also highlight the importance of early preventive interventions during adolescence and emerging adulthood. School-based mental health promotion programmes, early recognition and treatment of depression and other common mental disorders, anti-stigma initiatives, and accessible crisis intervention services may contribute to reducing suicide risk in young people. At the same time, targeted road safety education, particularly for newly licensed drivers, together with interventions addressing alcohol- and substance-related risk behaviours, may help reduce traffic-related mortality. Because many behavioural risk factors emerge during adolescence, preventive efforts implemented during this developmental period may yield substantial long-term public health benefits.

The pronounced summer peak in traffic accident mortality and the persistent regional inequalities identified in this study suggest that universal national strategies should be complemented by seasonally targeted and region-specific interventions. Strengthening road safety enforcement during high-risk summer months and improving access to mental health services in the Great Plain regions may contribute to further reductions in preventable mortality.

Further research using individual-level mortality records should examine method-specific suicide trends to better understand whether changes in the distribution of suicide methods have contributed to the observed decline in suicide mortality. Such analyses could provide important evidence for the development and refinement of targeted suicide prevention strategies.

### Limitations

The principal strengths of this study include the use of nationwide population-based mortality data covering a 25-year period and the simultaneous evaluation of the two leading external causes of death among Hungarian youth.

Several limitations should be considered when interpreting our findings. First, the ecological design based on aggregated population-level data precludes individual-level analysis of risk factors and does not allow causal inference. The possibility of ecological fallacy must therefore be acknowledged.

Second, although national mortality registers in Hungary are generally considered to be of high quality, some degree of misclassification of causes of death cannot be entirely excluded. As with all mortality registry studies, inaccuracies in death certification or coding may have affected a small proportion of cases.

Third, subtle changes in death certification practices or ICD-10 coding over the 25-year study period may have influenced temporal comparability, even in the absence of major classification revisions.

Fourth, monthly population denominators for the 15–24-year age group were estimated indirectly due to the absence of official monthly data. Although the extrapolation method provides reasonable approximations, minor inaccuracies may have influenced seasonal rate estimates.

Fifth, deaths of undetermined intent (ICD-10 Y10–Y34) were not included in the analyses. Although some studies have considered these deaths as potentially containing misclassified suicides, the HCSO database does not allow their separate extraction because they are available only within a broader aggregated category that also includes several other heterogeneous external-cause classifications. Consequently, a small degree of underestimation of suicide mortality cannot be excluded.

Finally, migration dynamics among young adults and potential heterogeneity within the youth population (e.g., socioeconomic or urban–rural differences) could not be examined in detail using aggregate data. Future research incorporating individual-level or mixed-method approaches would help to clarify underlying mechanisms.

## Conclusions

Between 2000 and 2024, mortality from external causes among Hungarian youth aged 15–24 years declined significantly; however, external causes remain the leading contributors to death in this age group. Persistent gender disparities, pronounced regional inequalities – particularly in the Great Plain regions – and a marked summer peak in traffic accident mortality highlight concentrated and preventable risk patterns.

The parallel assessment of suicide and traffic accident mortality provides a more comprehensive perspective on premature youth deaths and underscores the need for integrated prevention strategies. Sustained national efforts, combined with region-specific and seasonally targeted interventions, are essential to achieve further reductions in preventable mortality among young people in Hungary.

## Supplementary Information

Additional file 1.

Additional file 2.

## Data Availability

The data used in this study are available from: [https://statinfo.ksh.hu/Statinfo/themeSelector.jsp?&lang=en].

## References

[1] OECD, European Observatory on Health Systems and Policies, Hungary. Country Health Profile 2025. State of Health in the EU. Brussels: European Commission; 2025. https://health.ec.europa.eu/document/download/fff80b5a-c526-452a-857a-b15355dda60b_en.

[2] Hungarian Central Statistical Office. Dissemination database. 2024. https://statinfo.ksh.hu/Statinfo/themeSelector.jsp?⟨=en.

[3] World Health Organization. International Statistical Classification of Diseases and Related Health Problems, 10th Revision (ICD-10). Geneva: World Health Organization; 2019. https://icd.who.int/browse10/2019/en.

[4] Bertuccio P, Amerio A, Grande E, La Vecchia C, Costanza A, Aguglia A, Berardelli I, Serafini G, Amore M, Pompili M, Odone A. Global trends in youth suicide from 1990 to 2020: an analysis of data from the WHO mortality database. EClinicalMedicine. 2024;70:102506. 10.1016/j.eclinm.2024.102506. PMC10911948 38440131

[5] World Health Organization. Suicide worldwide in 2021: Global health estimates. Geneva: World Health Organization. 2025. https://iris.who.int/server/api/core/bitstreams/769d0a45-b50a-4b17-ba40-259bef44d9dd/content.

[6] Rihmer Z, Gonda X, Kapitany B, Dome P. Suicide in Hungary-epidemiological and clinical perspectives. Ann Gen Psychiatry. 2013;12(1):21. 10.1186/1744-859X-12-21. PMC3698008 23803500

[7] Rihmer Z, Dome P, Gonda X, et al. Decreasing suicide mortality in Hungary – What are the main causes? Eur Psychiatry. 2022;65(Suppl 1):S124. 10.1192/j.eurpsy.2022.342. Published 2022 Sep 1.

[8] European Commission. Young people. Facts and Figures. European Road Safety Observatory (ERSO). Brussels: European Commission, Directorate-General for Mobility and Transport. 2024. https://road-safety.transport.ec.europa.eu/document/download/8980a141-423e-4d2e-a083-f81c7e70ea00_en.

[9] Byrnes JP, Miller DC, Schafer WD. Gender differences in risk taking: a meta-analysis. Psychol Bull. 1999;125:367–83.

[10] Urfi Z, Nyári TA, Lantos T. Trends in childhood mortality from external causes of death in Hungary between 2000 and 2022: a retrospective cohort study of the nationwide population register data. BMJ Open. 2025;15:e099074. 10.1136/bmjopen-2025-099074. PMC12548609 41125268

[11] Eurostat. Revision of the European Standard Population: Report of Eurostat’s Task Force. Luxembourg: Publications Office of the European Union. 2013. https://ec.europa.eu/eurostat/documents/3859598/5926869/KS-RA-13-028-EN.PDF.

[12] Eurostat. Regions in the European Union: Nomenclature of Territorial Units for Statistics (NUTS) 2016/EU-28. Luxembourg: Publications Office of the European Union; 2018. 10.2785/022137.

[13] Moksony F. A Poisson-regresszió alkalmazása a szociológiai és demográfiai kutatásban. Demográfia. 2006;49(4):366–82.

[14] Kim HJ, Fay MP, Feuer EJ, Midthune DN. Permutation tests for joinpoint regression with applications to cancer rates. Stat Med. 2000;19(3):335–51. 10.1002/(sici)1097-0258(20000215)19:3<335::aid-sim336>3.0.co;2-z 10649300

[15] Edwards JH. The recognition and estimation of cyclic trends. Ann Hum Genet. 1961;25:83–7. 10.1111/j.1469-1809.1961.tb01501.x 13725808

[16] Walter SD, Elwood JM. A test for seasonality of events with a variable population at risk. Br J Prev Social Med. 1975;29(1):18–21. 10.1136/jech.29.1.18 PMC478881 1137765

[17] Diekstra RFW. The Prevention of Suicidal Behavior: Evidence for the Efficacy of Clinical and Community-based Programs International Journal of Mental Health, in Evaluation of Treatment Methods in Psychiatry. 1992;21:69–87. https://www.jstor.org/stable/41344636.

[18] World Health Organization. LIVE LIFE: an implementation guide for suicide prevention in countries. Geneva: World Health Organization; 2021. https://www.who.int/publications/i/item/9789240026629.

[19] Zonda T, Bozsonyi K, Veres E. Seasonal fluctuation of suicide in Hungary between 1970–2000. Archives Suicide Res. 2005;9(1):77–85. 10.1080/13811110590512967. 16040582

[20] Lantos T, McNally RJQ, Nyári TA. Patterns of suicide deaths in Hungary between 1995 and 2017. SSM Popul Health. 2021;16:100958. 10.1016/j.ssmph.2021.100958. PMC8592871 34815998

[21] Lantos T, Nyári TA, McNally RJQ. Seasonal variation of mortality from external causes in Hungary between 1995 and 2014. PLoS ONE. 2019;14(6):e0217979. 10.1371/journal.pone.0217979. PMC6553771 31170243

[22] Sebestyen B, Rihmer Z, Balint L, Szokontor N, Gonda X, Gyarmati B, Bodecs T, Sandor J. Gender differences in antidepressant use-related seasonality change in suicide mortality in Hungary, 1998–2006. World J Biol Psychiatry. 2010;11(3):579–85. 10.3109/15622970903397722. 20218927

[23] European Commission. Annual statistical report on road safety in the EU. 2025. European Road Safety Observatory. Brussels: European Commission, Directorate-General for Mobility and Transport; 2025. https://road-safety.transport.ec.europa.eu/european-road-safety-observatory/statistics-and-analysis-archive/annual-accident-report_en.

[24] Eurostat. Road safety statistics in the European Union. Statistics Explained. Luxembourg: Publications Office of the European Union; 2026. https://ec.europa.eu/eurostat/statistics-explained/index.php?title=Road_safety_statistics_in_the_EU.

[25] Balázs J, Miklósi M, Keresztény A, Hoven CW, Carli V, Wasserman C, Apter A, Bobes J, Brunner R, Cosman D, Cotter P, Haring C, Iosue M, Kaess M, Kahn JP, Keeley H, Marusic D, Postuvan V, Resch F, Saiz PA, Sisask M, Snir A, Tubiana A, Varnik A, Sarchiapone M, Wasserman D. Adolescent subthreshold depression and anxiety: psychopathology, functional impairment and increased suicide risk. J Child Psychol Psychiatry. 2013;54(6):670–7. 10.1111/jcpp.12016. 23330982

