# Supplementary material for: Trends and seasonal patterns in suicide and traffic accident mortality among youth in Hungary, 2000–2024: a nationwide population-based study
Source: BMC Pediatr. 2026 Jul 29;26:1149. doi: 10.1186/s12887-026-07419-9 (PMC13749969; doi:10.1186/s12887-026-07419-9)
Supplement: Supplementary file 1 [file 12887_2026_7419_MOESM1_ESM.docx]

**Table S1. Incidence rate ratios for deaths from external causes among youth, 2000–2024.**

| Subgroup | Youth deaths from external causes | | |
| --- | --- | --- | --- |
|  | Total (n=7209) IRR (95% CI) | Male (n=5700) IRR (95% CI) | Female (n=1509) IRR (95% CI) |
| GENDER |  |  |  |
| Female | 1.00 (Reference) | – | – |
| Male | 3.59 (3.39, 3.81) | – | – |
| AGE GROUP |  |  |  |
| 15–19 years | 1.00 (Reference) | 1.00 (Reference) | 1.00 (Reference) |
| 20–24 years | 1.57 (1.49, 1.64) | 1.71 (1.62, 1.81) | 1.14 (1.03, 1.26) |
| ANNUAL TREND (YEAR) | 0.97 (0.96, 0.97) | 0.97 (0.96, 0.97) | 0.97 (0.97, 0.98) |
| NUTS2 REGION |  |  |  |
| Budapest | 1.00 (Reference) | 1.00 (Reference) | 1.00 (Reference) |
| Pest County | 1.34 (1.22, 1.47) | 1.31 (1.18, 1.46) | 1.35 (1.11, 1.64) |
| Central Transdanubia | 1.29 (1.17, 1.43) | 1.27 (1.14, 1.42) | 1.26 (1.02, 1.54) |
| West. Transdanubia | 1.15 (1.04, 1.28) | 1.20 (1.06, 1.35) | 0.91 (0.72, 1.15) |
| South. Transdanubia | 1.39 (1.25, 1.53) | 1.44 (1.29, 1.62) | 1.11 (0.89, 1.39) |
| Northern Hungary | 1.42 (1.29, 1.56) | 1.45 (1.31, 1.61) | 1.23 (1.01, 1.50) |
| Northern Great Plain | 1.32 (1.21, 1.44) | 1.37 (1.24, 1.52) | 1.08 (0.89, 1.31) |
| Southern Great Plain | 1.50 (1.37, 1.64) | 1.51 (1.36, 1.68) | 1.38 (1.14, 1.67) |

*Notes:* Incidence rate ratios (IRRs) were estimated using negative binomial regression models with the logarithm of relevant population included as an offset term.

**Table S2. Incidence rate ratios for suicide deaths among youth, 2000–2024.**

| Subgroup | Youth suicide deaths | | |
| --- | --- | --- | --- |
|  | Total (n=2382) IRR (95% CI) | Male (n=1952) IRR (95% CI) | Female (n=430) IRR (95% CI) |
| GENDER |  |  |  |
| Female | 1.00 (Reference) | – | – |
| Male | 4.32 (3.89, 4.79) | – | – |
| AGE GROUP |  |  |  |
| 15–19 years | 1.00 (Reference) | 1.00 (Reference) | 1.00 (Reference) |
| 20–24 years | 1.69 (1.55, 1.84) | 1.90 (1.72, 2.08) | 1.03 (0.85, 1.25) |
| ANNUAL TREND (YEAR) | 0.98 (0.97, 0.98) | 0.98 (0.97, 0.98) | 0.99 (0.98, 1.00) |
| NUTS2 REGION |  |  |  |
| Budapest | 1.00 (Reference) | 1.00 (Reference) | 1.00 (Reference) |
| Pest County | 1.14 (0.97, 1.34) | 1.14 (0.94, 1.37) | 1.06 (0.75, 1.51) |
| Central Transdanubia | 1.08 (0.91, 1.28) | 1.07 (0.89, 1.30) | 1.02 (0.71, 1.47) |
| West. Transdanubia | 0.94 (0.78, 1.13) | 1.00 (0.81, 1.22) | 0.66 (0.43, 1.02) |
| South. Transdanubia | 1.20 (1.01, 1.43) | 1.25 (1.03, 1.51) | 0.95 (0.64, 1.40) |
| Northern Hungary | 1.39 (1.19, 1.62) | 1.48 (1.25, 1.75) | 0.97 (0.68, 1.39) |
| Northern Great Plain | 1.23 (1.06, 1.43) | 1.32 (1.12, 1.56) | 0.85 (0.61, 1.20) |
| Southern Great Plain | 1.40 (1.21, 1.63) | 1.47 (1.24, 1.74) | 1.10 (0.79, 1.55) |

*Notes:* Incidence rate ratios (IRRs) were estimated using negative binomial regression models with the logarithm of relevant population included as an offset term.

**Table S3. Incidence rate ratios for deaths from traffic accidents among youth, 2000–2024.**

| Subgroup | Youth deaths from traffic accidents | | |
| --- | --- | --- | --- |
|  | Total (n=2993) IRR (95% CI) | Male (n=2297) IRR (95% CI) | Female (n=696) IRR (95% CI) |
| GENDER |  |  |  |
| Female | 1.00 (Reference) | – | – |
| Male | 3.13 (2.88, 3.41) | – | – |
| AGE GROUP |  |  |  |
| 15–19 years | 1.00 (Reference) | 1.00 (Reference) | 1.00 (Reference) |
| 20–24 years | 1.64 (1.52, 1.76) | 1.79 (1.64, 1.96) | 1.23 (1.06, 1.43) |
| ANNUAL TREND (YEAR) | 0.95 (0.94, 0.95) | 0.95 (0.94, 0.95) | 0.96 (0.95, 0.97) |
| NUTS2 REGION |  |  |  |
| Budapest | 1.00 (Reference) | 1.00 (Reference) | 1.00 (Reference) |
| Pest County | 2.17 (1.84, 2.56) | 2.16 (1.79, 2.61) | 2.08 (1.50, 2.87) |
| Central Transdanubia | 2.10 (1.78, 2.48) | 2.07 (1.71, 2.52) | 2.04 (1.47, 2.84) |
| West. Transdanubia | 2.04 (1.72, 2.43) | 2.19 (1.80, 2.66) | 1.48 (1.03, 2.14) |
| South. Transdanubia | 2.23 (1.88, 2.65) | 2.33 (1.92, 2.84) | 1.84 (1.30, 2.61) |
| Northern Hungary | 1.73 (1.46, 2.04) | 1.72 (1.41, 2.09) | 1.66 (1.19, 2.32) |
| Northern Great Plain | 1.91 (1.63, 2.24) | 1.94 (1.62, 2.33) | 1.75 (1.28, 2.40) |
| Southern Great Plain | 2.36 (2.02, 2.77) | 2.41 (2.01, 2.90) | 2.13 (1.55, 2.92) |

*Notes:* Incidence rate ratios (IRRs) were estimated using negative binomial regression models with the logarithm of relevant population included as an offset term.
