# Supplementary material for: Trends and seasonal patterns in suicide and traffic accident mortality among youth in Hungary, 2000–2024: a nationwide population-based study
Source: BMC Pediatr. 2026 Jul 29;26:1149. doi: 10.1186/s12887-026-07419-9 (PMC13749969; doi:10.1186/s12887-026-07419-9)
Supplement: Supplementary file 2 [file 12887_2026_7419_MOESM2_ESM.docx]

**Supplementary Figure S1.** Temporal trends in male-to-female mortality rate ratios for all external causes (A), suicide (B), and traffic accidents (C) among individuals aged 15–24 years in Hungary, 2000–2024. Joinpoint regression lines, annual percent changes (APCs), and statistically significant joinpoints are shown.

**Supplementary Figure S2.** Sex-specific (males: left panels; females: right panels) geographical distribution of mortality from all external causes (A–B), suicide (C–D), and traffic accidents (E–F) among individuals aged 15–24 years in Hungary, 2000–2024. Maps show age-standardised mortality rates per 100,000 population based on the European Standard Population (ESP 2013).

**Supplementary Figure S3.** Sex-specific annual trends in mortality from all external causes (A), suicide (B), and traffic accidents (C) among individuals aged 15–24 years in Hungary, 2000–2024. Points represent annual mortality rates; solid lines represent fitted negative binomial regression trends with 95% confidence intervals.

**Supplementary Figure S4.** Sex-specific seasonal patterns of mortality from all external causes (A), suicide (B), and traffic accidents (C) among individuals aged 15–24 years in Hungary, 2000–2024. Points represent monthly mortality rates; curves represent fitted negative binomial regression seasonal trends with 95% confidence intervals.
